# Potential Adjuvant Therapeutic Effect of *Lactobacillus plantarum* Probio-88 Postbiotics against SARS-COV-2

**DOI:** 10.3390/vaccines9101067

**Published:** 2021-09-24

**Authors:** Irfan A. Rather, Sy-Bing Choi, Majid Rasool Kamli, Khalid Rehman Hakeem, Jamal S. M. Sabir, Yong-Ha Park, Yan-Yan Hor

**Affiliations:** 1 Department of Biological Sciences, Faculty of Science, King Abdulaziz University, Jeddah 21589, Saudi Arabia; mkamli@kau.edu.sa (M.R.K.); kur.hakeem@gmail.com (K.R.H.); jsabir2622@gmail.com (J.S.M.S.); 2 Centre for Bioinformatics, School of Data Sciences, Perdana University, Suite 9.2, 9th Floor, Wisma Chase Perdana, Changkat Semantan, Wilayah Persekutuan, Kuala Lumpur 50490, Malaysia; sybing.choi@perdanauniversity.edu.my; 3 Department of Biotechnology, Yeungnam University, 280 Daehak-ro, Gyeongsan 38541, Gyeongbuk-do, Korea; peter@yu.ac.kr; 4 Probionic Corporation Jeonbuk Institute for Food-Bioindustry, 111-18, Wonjangdong-gil, Deokjin-gu, Jeonju-si 54810, Jeollabuk-do, Korea; 5 PYH Lab, Yeungnam University, 280 Daehak-ro, Gyeongsan 38541, Gyeongbuk-do, Korea

**Keywords:** *Lactobacillus* *plantarum*, SARS-COV-2, COVID-19, postbiotics, plantaricin, helicase, viral replication, ROS, ERK, inflammatory

## Abstract

In response to the ongoing COVID-19 pandemic, the global effort to develop high efficacy countermeasures to control the infection are being conducted at full swing. While the efficacy of vaccines and coronavirus drugs are being tested, the microbiome approach represents an alternative pathophysiology-based approach to prevent the severity of the infection. In the current study, we evaluated the action of a novel probiotic *Lactobacillus* *plantarum* Probio-88 against SARS-COV-2 replication and immune regulation using an in vitro and in silico study. The results showed that extract from this strain (P88-CFS) significantly inhibited the replication of SARS-COV-2 and the production of reactive oxygen species (ROS) levels. Furthermore, compared with infected cells, P88-CFS treated cells showed a significant reduction in inflammatory markers such as IFN-α, IFN-β, and IL-6. Using an in silico molecular docking approach, it was postulated that the antiviral activity of *L. plantarum* Probio-88 was derived from plantaricin E (PlnE) and F (PlnF). The high binding affinity and formation of hydrogen bonding indicated that the association of PlnE and PlnF on SARS-COV-2 helicase might serve as a blocker by preventing the binding of ss-RNA during the replication of the virus. In conclusion, our study substantiated that P88-CFS could be used as an integrative therapeutic approach along with vaccine to contain the spread of the highly infectious pathogen and possibly its variants.

## 1. Introduction

Coronaviruses (COVs) have caused two large-scale pandemics in the past two decades, severe acute respiratory syndrome (SARS) and the Middle East respiratory syndrome (MERS) [1]. In 2019, a newly emerged, highly pathogenic human coronavirus—severe acute respiratory syndrome coronavirus 2 (SARS-COV-2) caused a major disease (COVID-19) outbreak, resulting in a pandemic declaration by the World Health Organization (WHO). Nearly half (40–45%) of the infected persons are asymptomatic, and infectivity usually peaks before the onset of the symptoms, which contributes to the difficulty in containing the disease spread [2,3]. Infection with COVID-19 can cause severe pneumonia due to an overactive immune system, triggering a cytokine storm which leads to respiratory distress and subsequent shutdown of multiple organs [4].

Considering the importance of immune regulation in the severity and progression of COVID-19, probiotic intervention has been suggested as a potential therapeutic agent in the event of SARS-COV-2 infections attributed to its immune modulation ability. By definition, “probiotics are live microorganisms that, when administered in adequate amounts, confer a health benefit to the host”. Probiotics are considered a nutraceutical, mainly comprised of lactic acid bacteria, and have a long history of use in food to stimulate the immune system, including increased resistance to infections. In a randomized, double-blind, placebo-controlled trial involving 100 children with severe sepsis, probiotic administration for seven days was shown to exert immunomodulatory effects. In the study, probiotics significantly decreased pro-inflammatory cytokines (IL-6, IL-12p70, IL-17, and TNF-α) while increasing anti-inflammatory (IL-10 and TGF-β1) cytokines [5]. This was further corroborated by a recent meta-analysis, showing that probiotic intervention reduced IL-6 and C-reactive protein levels in middle-aged and older adults with chronic low-grade inflammation [6]. Probiotics may potentially counterbalance the over-reactive inflammatory state caused by SARS-COV-2 infections through modulation of the innate and acquired immune systems, thereby preventing the deterioration of lung function.

The beneficial impact of microbiome modulation using probiotics in viral diseases has been demonstrated in numerous studies where some strains of lactobacilli and bifidobacteria have a protective role against influenza virus, rhinovirus, respiratory syncytial virus, adenovirus, and pneumovirus [7]. For decades, using probiotics as antiviral agents has been standard practice in aquaculture [8] as well as the poultry and swine industry. In our previous study, *Lactobacillus plantarum* Probio-38 and *L. salivarius* Probio-37 were found to exhibit antiviral activity against transmissible gastroenteritis coronavirus (TGEV) [9]. In a human clinical study involving 70 COVID-19 positive patients, oral bacteriotherapy consisting of multiple strains of lactic acid bacteria showed a statistically significant impact on the clinical conditions of COVID-19 patients [10]. Within 72 h, nearly all patients treated with bacteriotherapy showed remission of diarrhea and other symptoms compared to less than half of the not supplemented group. The estimated risk of developing respiratory failure was eight-fold lower in patients receiving oral bacteriotherapy. The prevalence of patients transferred to ICU and mortality was higher among the patients not treated with oral bacteriotherapy. The use of probiotics as an antiviral therapy against respiratory and enteric viruses has also been extensively reviewed as a new concept in medical science, shedding light on probiotics use against SARS-COV-2 [11]. This study aimed to evaluate the efficacy of probiotic metabolites in viral replication and immune regulation using in vitro and in silico studies

## 2. Materials and Methods

### 2.1. Bacterial Strains, Media, and Growth Conditions

The bacteria used in this study was *Lactobacillus plantarum* Probio-88, deposited with the Korean Collection for Type Cultures having the deposition number KCTC 14482BP. The strain was previously isolated from Kimchi, a Korean traditional fermented cabbage. A modified de Man–Rogosa–Sharpe (MRS) media containing 5 g/L soy peptone, 5 g/L sodium acetate, 20 g/L yeast extract, 20 g/L d (+)-glucose, 2 g/L ammonium sulfate, 2 g/L dipotassium phosphate, 0.1 g/L magnesium sulfate, 0.05 g/L manganese sulfate, and 1 g/L Tween 80 was used to culture the strain at 37 °C for 18 h. The strain was preserved in 25% glycerol (−45 °C) and activated three consecutive times using 1% inoculum before any experimental analysis. All chemicals herein, unless otherwise stated, were obtained from Sigma-Aldrich, St. Louis, MO, USA). 

### 2.2. SARS-COV-2 Virus

SARS-COV-2 strain was obtained from an oropharyngeal swab specimen of a patient with coronavirus disease 2019 (COVID-19). Any work with SARS-COV-2 was performed under biosafety level 3 (BSL-3) containment conditions. Virus stocks were generated by culturing the virus in HEK 293 cells at 90% confluency for 48 h. After that, cultures were aliquoted and stored at −80 °C. Control samples were prepared in the same manner using uninfected cells. Virus stocks were quantified by titration.

### 2.3. Preparation of Cell-Free Supernatant (CFS) from Lactobacillus plantarum Probio-88 (P88-CFS)

To produce CFS, *L. plantarum* Probio-88 was grown in modified MRS broth at 37 °C for 24 h, followed by centrifugation at 3500 rpm for 15 min. The resulting supernatant was collected, neutralized to pH 7.0, and filter-sterilized using a syringe filter (pore size 0.22 µm). After that, the supernatant was freeze-dried and reconstituted with water to 10% of its original volume. The resulting extract was coined as P88-CFS and stored at −20 °C for subsequent analysis.

### 2.4. In Vitro Inhibition of SARS-COV-2 Replication

HEK 293 cells were first seeded in 96-well plates (TPP, Trasadingen, Switzerland) at a density of 4 × 10^4^ cells per well. After 24 h, the cells were incubated with 100 μL culture medium for virus infection (DMEM supplemented with penicillin (100 U/mL) and streptomycin (100 μg/mL) + P88-CFS (50, 100, 150, 200 or 250 mg/mL) + SARS-COV-2 virus (0.1 multiplicity of infection). After 2 h of incubation at 37 °C, the cells were rinsed three times with PBS and maintained in 100 μL of the same culture medium in the absence of virus. The cells were incubated for another 48 h. Positive and negative control samples were inoculated in the same manner except that the P88-CFS was replaced with DMSO and phosphate-buffered saline (PBS), respectively. Viral DNA copies (viral load) was measured using the quantitative real-time PCR method.

### 2.5. Cell-Viability Assay

Human corneal epithelial (HCE), HEK 293, and Hela cell lines were cultured in complete medium (Dulbecco’s Modified Eagle’s Medium (DMEM) supplemented with 10% fetal bovine serum (FBS) (HyClone Laboratories, Logan, UT, USA), penicillin (100 U/mL), and streptomycin (100 μg/mL) and incubated at 37 °C in a humidified atmosphere of 5% CO_2_. Prior to MTT assay, the cells were seeded in 96-well plates at a density of 4 × 10^4^ cells per well. After 24 h, the cells were treated with DMEM containing different concentrations of P88-CFS (50, 100, 150, 200, or 250 mg/mL) for 48 h at 37 °C and 5% CO_2_. For the control, PBS was used to replaced P88-CFS in DMEM. At the end of incubation, the cells were washed in PBS, treated with 50 μL of 5 mg/mL 3-(4,5-dimethylthiazol-2-yl)-2,5-diphenyl-2H-tetrazolium bromide (MTT) solution and incubated for 45 min at 37 °C. The cell supernatants were discarded, and formazan crystals were dissolved in isopropanol, followed by absorbance measurement at 570 nm. All assays were performed in triplicate. Percent viability was defined as the relative absorbance of P88-CFS treated cells as compared to the control cells. 

### 2.6. Determination of Intracellular ROS

Upon treatment with P88-CFS, the intracellular ROS level in the SARS-COV-2 infected HEK 293 cells were measured using H_2_DCFDA oxidation method. Briefly, media was removed, and the cells were washed twice with DMEM only. Cells were then incubated with 10 µM 2′-7′-dichlorodihydrofluorescein diacetate (H_2_DCFDA, Sigma Aldrich) in DMEM for 30 min at 37 °C. Following that, cells were washed twice with PBS, and the fluorescence intensity in H_2_DCFDA treated cells was determined by fluorescence microscopy.

### 2.7. Immunocytochemistry of Hela Cells

HEK293 cells were cultured in complete culture media and then infected with SARS-CoV-2. The cells were then washed three times in PBS and fixed in 4% formaldehyde for 10 min. Next, cells were subsequently permeabilized using 0.2% Triton X. Following that, the cells were treated with signal enhancer and then blocked using 5% normal goat serum diluted in PBS. Cells were incubated at 4 °C with a primary antibody of pERK (1:100) (Cell Signaling, Danvers, MA, USA) for 12 h. Cells were then washed with PBS three times and incubated with secondary antibodies for 1 h. The cells were then rinsed with PBS, and nuclei were counterstained with DAPI (Sigma Aldrich, St. Louis, MO, USA). Finally, cells were washed with PBS, and pictures were taken using a fluorescent microscope equipped with a digital camera (Nikon, Tokyo, Japan).

### 2.8. Quantitative Real-Time PCR

Total RNA was extracted from the HEK293 cells using Trizol reagent (Invitrogen, Waltham, MA, USA) as per the manufacturer’s instruction. The RNA concentration was measured using NanoDrop, and RNA was stored in diethyl pyrocarbonate-treated water at −80 °C. Further, 1 µg of RNA in a reaction mixture with a total volume of 25 µL was primed with random primers (Promega) and then reverse-transcribed at 72 °C for 5 min and 37 °C for 60 min. Real-time PCR was performed with 2 μL of cDNA, 10 pmol of each gene-specific primer, and Power SYBR^®^ Green PCR Master Mix (Applied Biosystems, Foster city, CA, USA) on a 7500 real-time PCR system (Applied Biosystems, Foster city, CA, USA). Detailed information describing the primer sequences for rt-PCR is provided in Table 1.

### 2.9. Antiviral Activity of Lactobacillus plantarum Probio-88 Using In Silico Molecular Docking 

#### Modelling of Plantaricin E and Plantaricin F

To construct the 3D structure for both plantaricin E and plantaricin F from *L. plantarum* Probio-88, the primary sequences were subjected to BLASTP (https://blast.ncbi.nlm.nih.gov/Blast.cgi, accessed on 30 April 2021) to search for sequence identity and to identify the appropriate template for homology modeling. Due to the high percentage of sequence identity with target sequences plantaricin E and F, a comparative modeling approach was adopted using bacteriocin plantaricin E (PlnE) and F (PlnF) (PDB ID:2JUI and 2RLW) as the template. An automated protein structure prediction server, SWISS-MODEL, was used, and the model with the highest QMEAN score was selected [12]. The structural assessment of the built model was conducted using Ramachandran plot analysis. 

HADDOCK2.4, an automated protein–protein docking server (https://wenmr.science.uu.nl/, accessed on 7 August 2021), was used to investigate protein–protein interaction of plantaricin E and F from *L. plantarum* P88 towards SARS-COV-2 helicase [13]. Helicase nsp13 from SARS-COV-2 (PDB ID: 6ZSL) was selected. Interacting residues for ATP and ss-RNA binding site namely Glu537, Arg567, Arg443, His290, Arg442, Asn265, Gly439, Lys288, Ser485, Lys146, Lys139, Tyr180, His230, Tyr198, Arg212, Pro335, Arg339, Asn516 were determined and set as the interface residues for docking [14]. The cluster with the least HADDOCK score was selected and further analyzed for binding using PRODIGY [15]. To visualize the binding interactions, Visual Molecular Dynamics (VMD) was used [16].

## 3. Results

**Figure 1:**
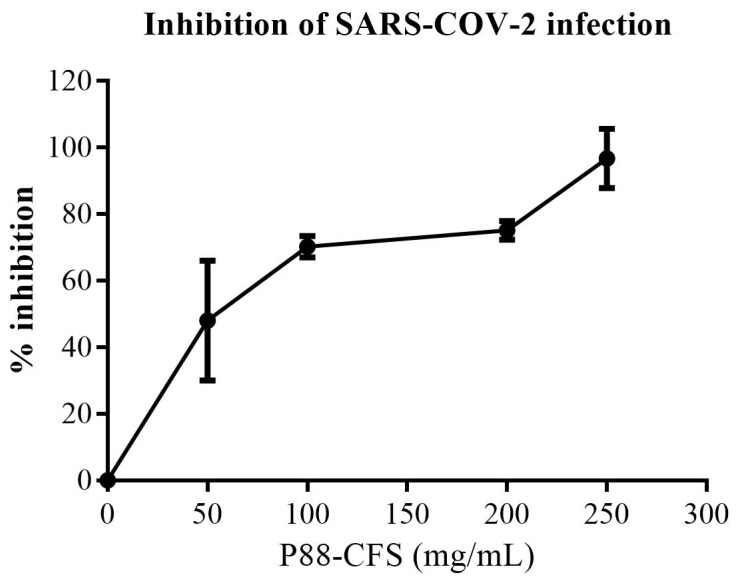
Inhibition of late-stage viral infection in HEK293. After 2 h p.i., DMSO or P88-CFS was added. Control samples were treated in the same manner with the same volume of DMSO. Data are represented as the means ± SD of 3 independent experiments.

**Figure 2:**
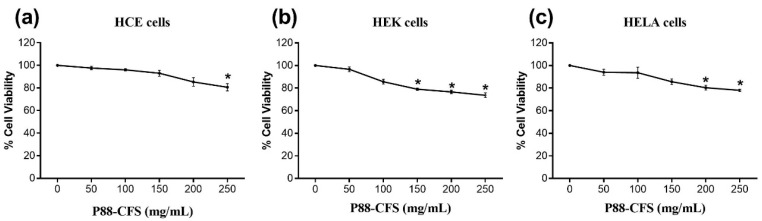
Percentage viability of (**a**) HCE, (**b**) HEK, and (**c**) HeLa cells treated with different concentrations of P88-CFS. MTT assay was performed to determine the percentage of cell viability in the presence of P88-CFS. Cells were seeded in 96 well plates at a density of 4 × 10^4^ and treated with P88-CFS for 24 h. Data are represented as the means ± SD of 3 independent experiments. * *p* < 0.05 compared with Control.

The inhibitory effect of P88-CFS was initially assessed in HEK 293 cells, where the cells were first infected with the SARS-COV-2 virus and then treated with P88-CFS at concentrations ranging from 50.0 to 250.0 mg/mL. Based on viral RNA copies determined by qPCR, the viral load in the culture supernatant was reduced compared to the untreated SARS-COV-2-infected control (0 mg/mL P88-CFS), as shown in Figure 1. The viral growth was significantly inhibited from 48% to 97%, with an increasing concentration of P88-CFS. Using MTT assay for cell cytotoxicity, the P88-CFS tested on HCE, HEK, and HeLa cell lines showed that the metabolite was well tolerated by the human cells. According to the guideline on the extent of cytotoxicity (ISO 10993-5), cell viability above 80% is deemed as non-cytotoxic, 80–60% is considered weak, and below 40% as strong cytotoxicity. HCE cells showed the most robust tolerance towards P88-CFS, followed by HeLa and HEK cells (Figure 2). Above 80% of cell viability was maintained in HCE and HeLa cells when treated with 200 mg/mL of P88-CFS. However, HEK cells showed higher sensitivity to the extract, as culture medium containing above 100 mg/mL P88-CFS resulted in less than 80% cell viability. The results reinforce that P88-CFS could inhibit the replication of highly infectious human coronavirus without any serious cytotoxicity effect if administered at 100 mg/mL; hence the same dosage was used for all subsequent experiments.

**Figure 3:**
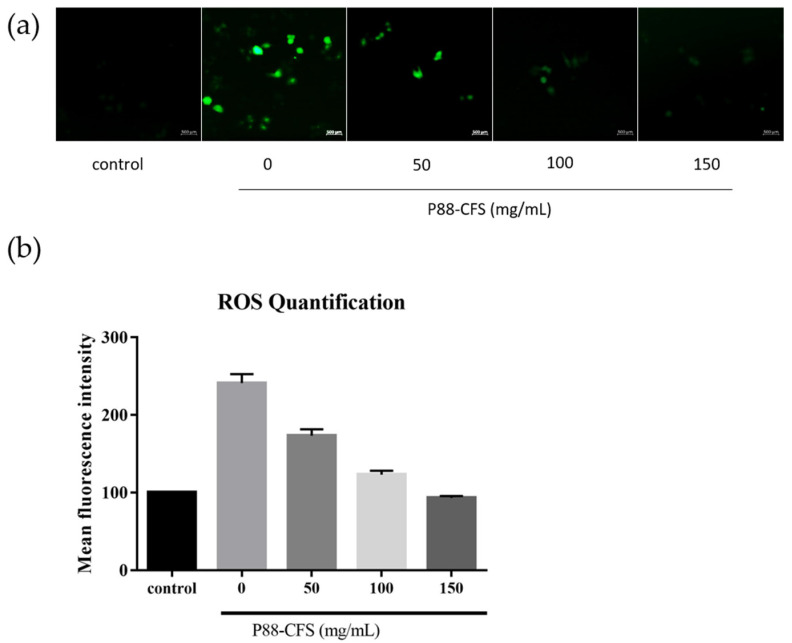
Reactive oxygen species (ROS) staining by dichlorofluorescein (H2DCFDA) stain (**a**) and quantitative fluorescence intensity measured by image J (**b**). Treatment cells were stained with H2DCFDA stain for 30 min and washed with PBS followed by fluorescence imaging under a confocal microscope [Scale is 500 µm].

We further investigated the effects of P88-CFS on the intracellular accumulation of reactive oxidizing species (ROS). Of note, ROS plays a critical role in the pathogenesis of COVID-19, where overaccumulation of ROS can worsen the disease severity. A significant increase in ROS levels was seen upon infecting cells with SARS-COV-2. However, when the infected cells were incubated with 50, 100, and 150 mg/mL P88-CFS, ROS levels were significantly reduced to approximately 1.7-fold, 1.2-fold, and 0.9-fold, respectively (Figure 3). 

**Figure 4:**
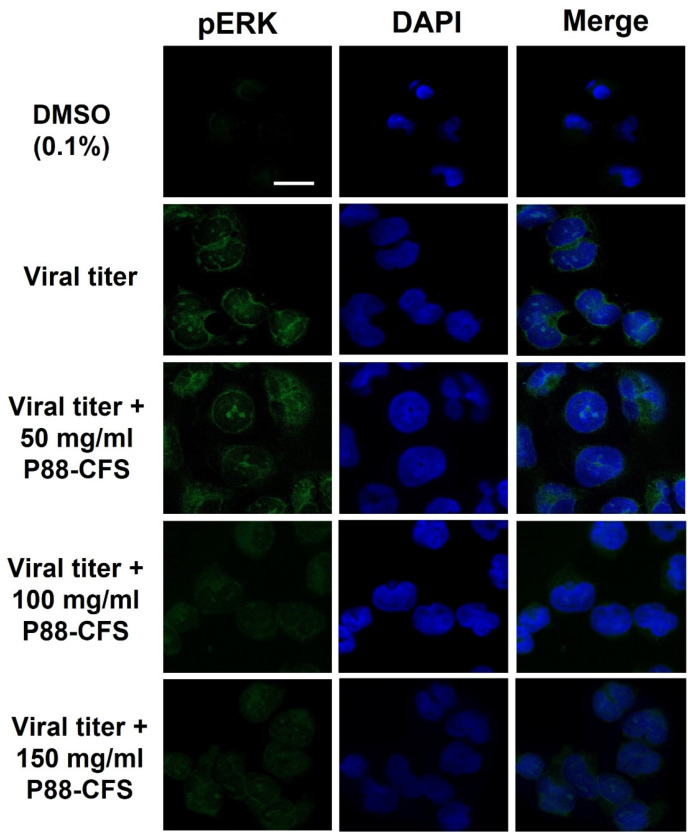
The influence of P88-CFS on p-ERK protein in HEK293 cells after infection with SARS-COV-2. Immunofluorescence was observed using confocal microscopy. The p-ERK expression in SARS-COV-2 infected HEK293 cells was treated with 50, 100 and 150 mg/mL of P88-CFS. DMSO (0.1%) was used as solvent for P88-CFS in all treatment groups. Cells were fixed 12 h post infection and confocal images were collected. Cell nuclei are denoted in blue, p-ERK is denoted in green [Scale bar = 5 μm].

The MAPK/ERK pathway is an important passage used by many viruses for its pathogenesis. Phosphorylated ERK (p-ERK) are often associated with the presence of viral infections. As shown in Figure 4, in virus-infected HEK 293 cells without any treatment, the expression of p-ERK was extensively observed in both the cell nucleus and cytoplasm, while this was nearly non-detectable in mock cells treated with 0.1% DMSO. Although 50 mg/mL of P88-CFS did not block the expression of p-ERK, the treatment with 100 and 150 mg/mL of P88-CFS drastically reduced the phosphorylation of ERK in SARS-COV-2 infected cells. 

**Figure 5:**
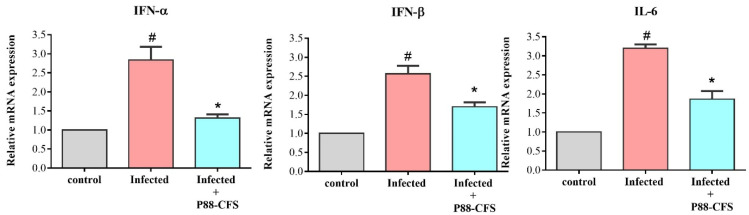
Gene expression of inflammatory markers in HEK 293 cells post infection with and without treatment of P88-CFS. The expression of IFN-α, IFN-β, and IL-6 were measured by qPCR and normalized to GAPDH. All the experiments were done in triplicate and statistical analysis was performed by *t*-test. Data are represented as the means ± SD of 3 independent experiments. * *p* < 0.05 compared with virus-infected cells; # *p* < 0.05 compared with control.

Finally, to assess the relation of virus infections with inflammation, IFN-α, IFN-β, and IL-6 were among the proinflammatory cytokines used in the study. In the SARS-COV-2 infected cells, the mRNA expression of IFN-α, IFN-β, and IL-6 increased significantly compared to control (Figure 5). Whereas in cells simultaneously infected with SARS-COV-2 and treated with 100 mg/mL P88-CFS, the mRNA expression levels of these proinflammatory cytokines decreased hugely. Compared to the infected cells, P88-CFS treated cells showed a 2.2-fold, 1.5-fold, and a 1.7-fold significant reduction in IFN-α, IFN-β, and IL-6, respectively. Such findings suggest that P88-CFS exhibits antiviral activity and regulates inflammatory responses caused by the virus invasion to the cells.

**Figure 6:**
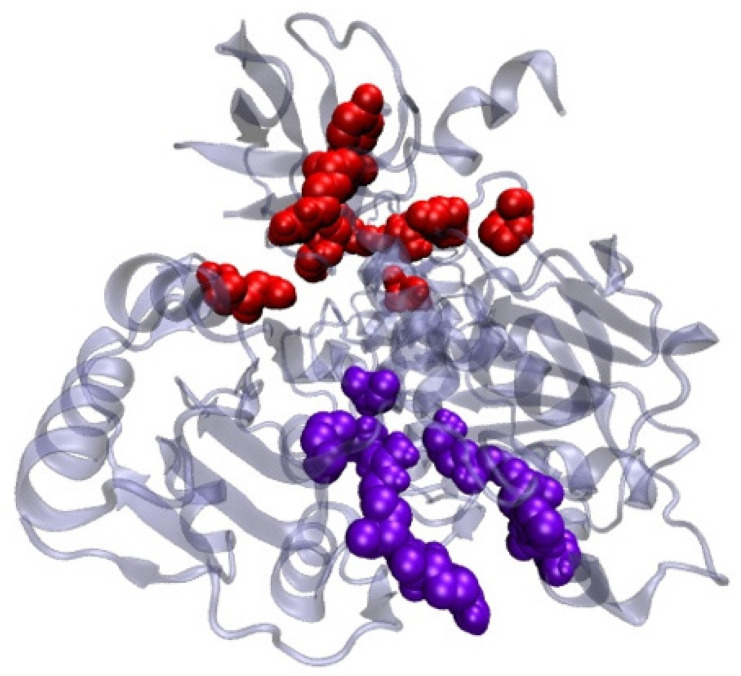
SARS-COV-2 Helicase nsp13 represented in secondary structure representation. The interacting residues for both ss-RNA and ATP binding are in red (Ser485, Lys146, Lys139, Tyr180, His230, Tyr198, Arg212, Pro335, Arg339, Asn516) and violet (Glu537, Arg567, Arg443, His290, Arg442, Asn265, Gly439, Lys288) respectively.

**Figure 7:**
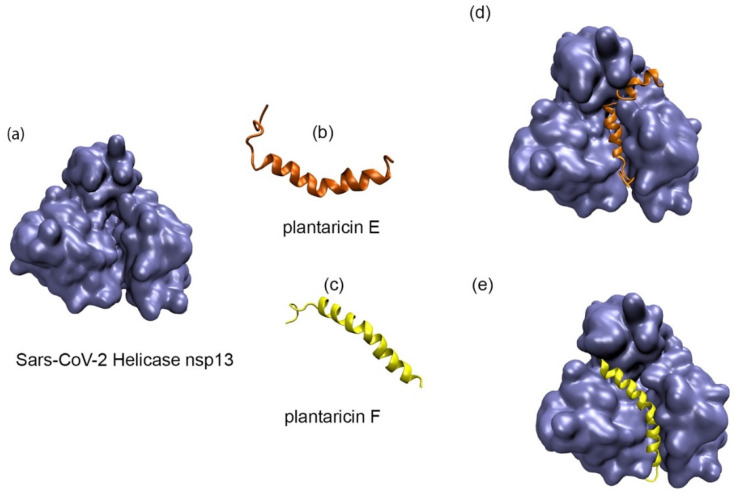
Molecular Docking of PlnE and PlnF. (**a**) The 3D structure of SARS-COV-2 helicase nsp 13 (PDB ID: 6ZSL) was used in the molecular docking studies. The built model for PlnE (**b**) and PlnF (**c**). The docked results shown that PlnE (**d**) and PlnF (**e**) were tucked into both the ss-RNA and the ATP binding site cavity.

Using an in silico molecular docking approach, the antiviral activity of *L. plantarum* Probio-88 was further evaluated. Based on the whole genome sequence, the strain was found to produce bacteriocin PlnE and PlnF, with 33 and 34 amino acid residues, respectively. The built model of PlnE and PlnF is made up of a single helix chain, and the backbone conformation was assessed using Ramachandran plot by measuring the psi (Ψ) and phi (Φ) angles of the backbone [17]. The Ramachandran plot statistical analysis reflected that 84% of the residues from PlnE and 92.9% from the PlnF built model lay in the most favorable region (Table 2 and Figure 6). The results correlated well and above the quality as compared to the template’s structure, which was elucidated using the NMR method 2JUI and 2RLW. The binding affinity of both plantaricin E and F against SARS-COV-2 helicase nsp13 was evaluated using the protein–protein docking approach. The main function of helicase involved the separation process of self-annealed ss-RNA using ATP as the energy source. Therefore, the surrounding residues at the ss-RNA and ATP binding sites serving as the active residues were applied as the HADDOCK protein–protein docking binding site parameter (Figure 6). Due to the long-extended helix structure in both PlnE and PlnF, the binding of PlnE and F toward SARS-COV-2 helicase nsp13 spanned across the ss-RNA and ATP binding sites with binding affinity of −17.4 kcal/mol and −15.6 kcal/mol, respectively (Figure 7, Table 3). 

**Figure 8:**
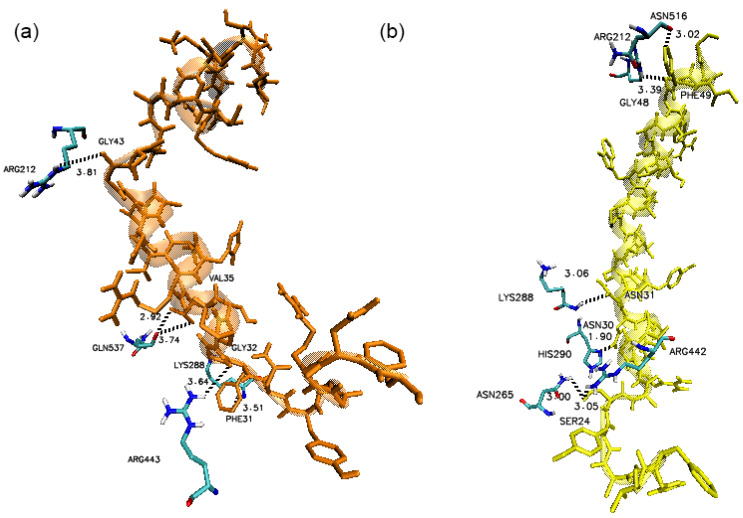
Binding conformation of (**a**) PlnE and (**b**) PlnF. Hydrogen bonding cut-off is set at 4.0 Å.

**Figure 9:**
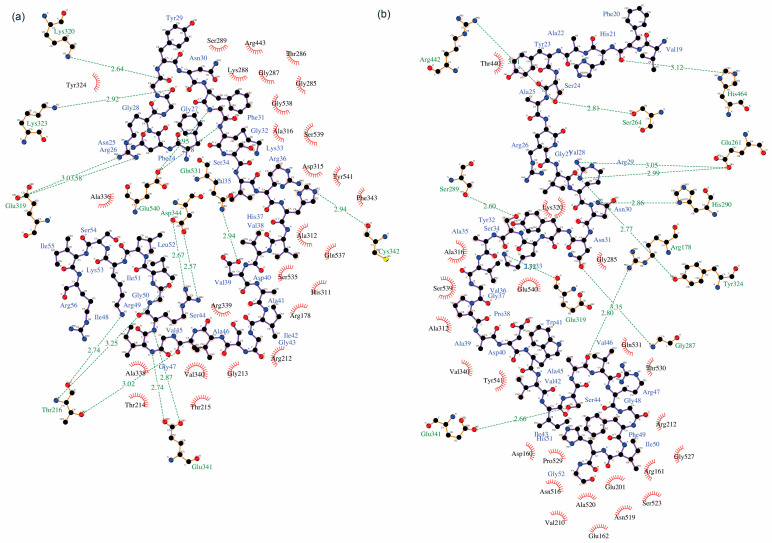
Ligplot + interaction diagram of (**a**) PlnE and (**b**) PlnF where hydrogen bonding is represented by the green dotted line and hydrophobic interaction represented in red.

The docking results revealed that the N-terminal of PlnE and PlnF were bound at the ATP binding sites whereas the C-terminals of PlnE and PlnF were attached to the ss-RNA binding site of helicase nsp 13. The residues at the C-terminal of PlnE, such as Val35, Gly32, and Phe31, formed several hydrogen bonds with Gln537, Arg443, and Lys 288 at the ATP binding site of SARS-COV-2 nsp 13 (Figure 8a). Newman and co-workers recently reported Gln537 and Arg443 as a crucial binding site for ATP hydrolysis [18]. In addition, the Gly43 located at the N-terminal of PlnE also formed a hydrogen bond interaction with one of the ss-RNA interfacing residues, namely Arg212 of SARS-COV-2 nsp 13 (Figure 8a). Similarly, for PlnF, hydrogen bond formation was present between Asn30 with His290 and Lys288 of SARS-COV-2 nsp13, Ser24 with Arg442, and Asn265 of SARS-COV-2 nsp13 (Figure 8b). Gly48 and Phe49 also occupied the ss-RNA binding site of SARS-COV-2 nsp12 at the N-terminal of PlnF by forming two hydrogen bonds with Arg212 and Asn516 (Figure 8b). Hydrophobic interactions, which are known to be the major contributions in protein–protein interaction, were also dominated in both PlnE and F interaction with the SARS-COV-2 nsp13 helicase (Figure 9).

## 4. Discussion

Most viruses, including coronavirus, utilize similar mechanisms to hijack host cells as replication machinery to produce their progeny. The process generally involves attachment and entry, translation of replicas, and assembly of the replication transcription complex, followed by genome replication and transcription, translation of structural proteins, and finally, virion assembly and release [19]. Host cells are also equipped with innate and adaptive immune responses to limit viral proliferation as a countermeasure. Ultimately, it becomes a race between the host and virus to determine the outcome of a disease progression [20]. 

Naturally, the host cell’s defense mechanisms are initiated spontaneously with the activation of phagocytes upon detection of virus entry. As phagocytes become activated, they release ROS, and accumulating evidence suggests that ROS also plays a crucial role in the pathogenesis of COVID-19 [21]. While ROS is well known for its deleterious effects, it is also a part of the important mediators vital for normal cellular function. The inability to regulate the overproduction of ROS during the viral infection can worsen the disease severity. An elevated level of ROS is also known to upregulate the expression of pro-oxidant cytokines such as tumor necrosis factor (TNF) and interleukin-1 cytokines, which ultimately will further increase ROS generation. This repeated cycle eventually causes a cytokine storm that leads to tissue damage and can be life-threatening. Excess ROS is also the leading cause of lung injury in other respiratory viral infections such as influenza virus infection [22]. Thus, it is worth noting that regulating ROS levels is a necessary process that drives pathological host responses against viral infection. 

The increased level of ROS during viral infection has also been reported to activate the ERK (extracellular signal-regulated kinase) pathway that is important in the pathogenesis of human coronavirus. In addition to ROS, the virus or its components can also activate the kinase. Mizutani et al. reported that Vero E6 cells infected with SARS-CoV showed enhanced ERK phosphorylation. In contrast, another study by Chen reported that SARS-CoV S protein or SARS-CoV virus-like particles could also phosphorylate ERK in A549 cells [23]. Similarly, MERS and HCoV-299E also modulated the ERK signaling pathway during infection [19]. The ERK pathway is composed of three signal cascades (Raf/MEK/ERK) and is crucial for cell growth. Its pathway activation is highly dynamic, thus becoming a target of exploitation by many viruses (herpes simplex type-1 virus, influenza virus, Ebola virus, flavivirus, and many others) for different purposes, including viral transcription, particle production, replication, as well as infectivity [24]. Indeed, it was also shown in our study that SARS-COV-2 activated ERK upon infecting HeLa cells.

Besides fulfilling viral-supportive roles, the ERK pathways are also crucial for the maintenance of inflammation amid infections. Initiation and modulation of inflammatory responses are one of the key events controlled by mitogen-activated protein kinases (MAPKs). The ERK pathway is one of the three members of the MAPKs family, along with p38 and JNK [25]. The MAPKs are activated upon viral invasion leading to the production of inflammatory cytokines and chemokines. In a highly pathogenic virus-like SARS-COV-2, a dysregulated inflammatory response that occurs upon pathogen challenge is termed a cytokine storm and is strongly controlled by the p38 and ERK pathways [25]. 

Inflammation during an infection represents a protective mechanism to contain pathogen spread. The first line of defense typically involves type 1 interferon (IFN-α and IFN-β) and multifunctional cytokine IL-6 [25]. In our present study, gene expression of IFN- α, IFN- β, and IL-6 were significantly increased when infected with SARS-COV-2 and the expression of these genes was lower in the presence of P88-CFS. While many studies showed that the SARS-COV-2 was able to dampen the IFN response, recent findings showed that IFN production is indeed being stimulated at a substantial level albeit delayed [26]. The group also reported that large amounts of viral transcripts were observed before the IFN induction in SARS-COV-2-infected cells. It is thus postulated that a high viral load is required to initiate IFN response. This finding was consistent with the observation that high viral loads were detected in COVID-19 cases soon after symptom onset [27]. We hypothesized that the lowered expression of these genes may be attributed to the lower amount of viral proliferation in the cells after being treated with P88-CFS.

In the present work, the treatment of SARS-COV-2 infected cells with P88-CFS managed to eradicate most of the virus post-infection, and the effect was found to be more pronounced with the increasing concentration of the P88-CFS. Consistent with a reduction of viral copies, P88-CFS also reduced inflammation and ERK activation, which are important cascading evidence of viral invasions. It is vital to acknowledge that clearance of inflammatory factors following inhibition of viral replication in cells could also prevent the cytokine storm, the deadliest of all in COVID-19 patients. 

Probiotics are known to secrete beneficial metabolites termed as postbiotics which could promote immunomodulatory effects and antibacterial and antiviral mechanisms; thus, it is unsurprising that probiotics were advocated as potential adjuvant therapy or prevention against SARS-COV-2 infection [28,29,30,31,32,33]. Some postbiotics, including bacteriocins have been proven to migrate across human epithelial cells to exert therapeutic effects [34]. The human respiratory tract is covered with mucosal epithelial surfaces that become the primary path for internalizing respiratory virus, including SAR-CoV-2 [35]. Therefore, targeting the epithelial surfaces would be an effective way of curbing viral infections. This could be made feasible by developing functional food or therapeutic products such as oronasal spray incorporated with postbiotics proven with antiviral activities. In the gut, the metabolites produced by probiotics can also penetrate the gastrointestinal epithelial surfaces and enter the bloodstream to reach targeted organs such as the lungs during a respiratory infection [36]. It is crucial for the interaction between gut microbes and the host’s immune system to respond to infections—the gut lung axis [36]. Therefore, both probiotics and postbiotics can serve as new horizons in microbial biotherapy with high versatility. 

Attention had been drawn to fermented cabbage products such as Kimchi, as an important dietary practice helpful in mitigating COVID-19 severity, in parallel with the relatively low COVID-19 death rate in countries such as Korea [37]. Besides modulating the antiviral immune response in respiratory and gastrointestinal mucosal tissue, *L. plantarum*, one of the abundant species found in Kimchi was also reported to produce bacteriocins (plantaricin) as antiviral compounds against several respiratory pathogens [29,38,39]. Based on a separate in silico study, Plantaricin W, D and JLA-9 were also found to have high inhibition against the catalytic site of RNA-dependent RNA polymerase of SARS-COV-2, which plays a crucial role in the replication cycle of the virus [28]. Similarly, our *L. plantarum* Probio-88 also demonstrated antiviral activity using both PlnE and PlnF in an in silico molecular docking approach. The high binding affinity and formation of hydrogen bonding indicated that the association of PlnE and PlnF on helicase might serve as a blocker by preventing the binding of ss-RNA on helicase. The utilization of ATP by helicase for the unwinding process might fail as the ATP binding site is occupied by the C-terminal of the plantaricins. Besides hydrophobic interactions, one of the major contributions in protein–protein interaction was also dominated in both PlnE and PlnF interaction with the helicase. This might not directly infer the binding of plantaricins towards helicase, but it possibly maintains the protein binding stability [40]. 

Moreover, the authors postulated that the direct anti-SARS-COV-2 replication activity of P88-CFS could have stemmed from the concoctions of postbiotics consisting of PlnE and PlnF, which work by preventing the helicase activities essential for viral genome replication, transcription, and translation [41]. The infectivity of coronaviruses depends on the spike protein which facilitates the viral attachment and entry to the host cells [42]. However, there is great variability in the structure of the spike proteins among coronavirus species, more so, the several variants of the SARS-COV-2 itself due to genetic mutations [43,44]. Conversely, helicase nsp13 is a non-structural protein highly conserved among all coronavirus species [44]. Thus, therapeutics targeting this enzyme would be more applicable to SARS-COV-2, its variants, and the closely related coronavirus species such as SARS-CoV and MERS-CoV. This may indicate that *L*. *plantarum* Probio-88 could be protective against variant strains of SARS-COV-2 [45]. Nevertheless, it is too early to conclude the exact mechanisms of the strain. Future research related to unravelling details of the molecular signaling is much needed, as is clinical validation. Although the current work is only preliminary, the antiviral effects demonstrated are substantial. We envision the potential to test P88-CFS on patients with early COVID-19 infection as adjuvant therapy to enhance existing vaccine-specific immune response for better control of the disease and encourage accelerated recovery.

## 5. Conclusions

The SARS-COV-2 inhibitory activity demonstrated with *L. plantarum* Probio-88 proved that probiotics could be used as an adjunct approach along with vaccine and other antiviral drugs to reduce the spread of the highly infectious pathogen. In addition, the strain exhibited potent anti-inflammatory activities, which could be very helpful in COVID-19 patients to prevent overactive inflammatory responses upon viral infection. Importantly, natural supplements like probiotics have been proven safe for decades of use with many other promising health benefits. The findings of this work support a valuable insight that *L. plantarum* Probio-88 could be a promising candidate to prevent the worsening of COVID-19 infection.

## Figures and Tables

**Table 1:** List of primers used in RT-PCR.

Target	Primer Directions	Sequences (5→3)
GAPDH	Forward Reverse	CAC CAC CAA CTG CTT AGC AC CCC TGT TGC TGT AGC CAA AT
IFN α	Forward Reverse	GAT GGC AAC CAG TTC CAG AAG AAA GAG GTT GAA GAT CTG CTG GAT
IFN β	Forward Reverse	CTC CAC TAC AGC TCT TTC CAT GTC AAA GTT CAT CCT GTC CTT
IL-6	Forward Reverse	AAC TCC TTC TCC AGA AGC GCC GTG GGG CGG CTA CAT CTT T

**Table 2:** The comparison of Ramachandran plot analysis for the plantaricin E and F built model and templates.

Region of Plot	Plantaricin E				Plantaricin F			
	Built Model		Template (PDB ID: 2JUI)		Built Model		Template (PDB ID: 2RLW)	
	No. of Residues	%	No. of Residues	%	No. of Residues	%	No. of Residues	%
Residue in most favoured region	21	84.0	21	84.0	26	92.9	25	89.3
Residue in additionally allowed region	4	16.0	4	16.0	1	3.6	2	7.1
Residue in generously allowed regions	0	0.0	0	0.0	1	3.6	1	3.6
Residue in disallowed region	0	0.0	0	0.0	0	0.0	0	0.0
Total	25	100	25	100	28	100	34	100

**Table 3:** Summary of molecular docking result, number of interactions involved for PlnE and PlnF.

Analysis	Types of Plantaricin	
	E	F
Binding Interaction		
∆G, (kcal/mol)	−17.4	−15.6
K_d_ (M) at 37 °C	5.8 × 10^−13^	1.0 × 10^−11^
Number of interactions		
Hydrogen Bonding	15	12
Hydrophobics	16	23

## Data Availability

The data available has been presented in the paper.
